# Directing reaction pathways via in situ control of active site geometries in PdAu single-atom alloy catalysts

**DOI:** 10.1038/s41467-021-21555-z

**Published:** 2021-03-09

**Authors:** Mengyao Ouyang, Konstantinos G. Papanikolaou, Alexey Boubnov, Adam S. Hoffman, Georgios Giannakakis, Simon R. Bare, Michail Stamatakis, Maria Flytzani-Stephanopoulos, E. Charles H. Sykes

**Affiliations:** 1 Department of Chemical and Biological Engineering, Tufts University, Medford, MA USA; 2 Thomas Young Centre and Department of Chemical Engineering, University College London, London, UK; 3 Stanford Synchrotron Radiation Light Source, SLAC National Accelerator Laboratory, Menlo Park, CA USA; 4 Department of Chemistry, Tufts University, Medford, MA USA

**Keywords:** Catalytic mechanisms, Heterogeneous catalysis, Chemical engineering, Atomistic models

## Abstract

The atomic scale structure of the active sites in heterogeneous catalysts is central to their reactivity and selectivity. Therefore, understanding active site stability and evolution under different reaction conditions is key to the design of efficient and robust catalysts. Herein we describe theoretical calculations which predict that carbon monoxide can be used to stabilize different active site geometries in bimetallic alloys and then demonstrate experimentally that the same PdAu bimetallic catalyst can be transitioned between a single-atom alloy and a Pd cluster phase. Each state of the catalyst exhibits distinct selectivity for the dehydrogenation of ethanol reaction with the single-atom alloy phase exhibiting high selectivity to acetaldehyde and hydrogen versus a range of products from Pd clusters. First-principles based Monte Carlo calculations explain the origin of this active site ensemble size tuning effect, and this work serves as a demonstration of what should be a general phenomenon that enables in situ control over catalyst selectivity.

## Abstract (web summary)

Single-atom alloys are promising catalysts for a number of different reactions. Here, the authors demonstrate that carbon monoxide can be used to transition a PdAu catalyst between a single atom and a cluster phase which exhibit distinct selectivities for ethanol dehydrogenation.

## Introduction

Bimetallic catalysts often exhibit enhanced catalytic performance compared to their monometallic counterparts and are widely used in many chemical transformations including reforming^1,2^, coupling^3^, pollution control^4,5^, and oxidation^6,7^ reactions. The specific reactivity of bimetallic catalysts is generally explained via ensemble (geometric) and/or ligand (electronic) effects that lead to enhanced reactivity and/or selectivity^8–10^. An extreme example of the ensemble effect is utilized in single-atom alloy (SAA) catalysts, in which a more reactive element is atomically dispersed in a less active host metal^11,12^. Usually, platinum group metals (PGM) are dispersed as isolated single atoms in coinage metals (Cu, Au, Ag) and they exhibit enhanced reactivity/selectivity and stability over their monometallic counterparts^13^. For example, the PtCu SAA is more efficient at alkane dehydrogenation than Cu while avoiding the coking that occurs on catalysts with extended Pt clusters^14^. This PtCu/SiO_2_ SAA is also highly selective for the hydrogenation of 1,3-butadiene to butene^15^ and acetylene to ethylene^14^ via a mechanism in which H_2_ dissociates at isolated Pt atom sites and the H atoms spill over to Cu, where the selective hydrogenation occurs. In contrast, Pt clusters lead to over-hydrogenation and deactivation. For some other reactions, clusters of the catalytically active metal atom are needed to activate chemical bonds and obtain the desired products. For example, contiguous Pd sites are necessary for O_2_ activation in the CO oxidation reaction, whereas single Pd atoms in Au are inactive for this reaction^16,17^. Also, Pd nanoparticles (NPs) are needed for the methane oxidation reaction, and the decomposition of the NPs into single Pd atoms at high temperatures leads to a drop in reactivity^18^. Furthermore, Ni and Pt clusters are required for the complete hydrogenation of ethylene to ethane, as this reaction does not take place on highly dilute NiCu alloys^19^ or the PtCu SAA^14^. For alcohol conversion, which is the focus of this study, isolated Ni atoms are not able to break the C–C bond of ethanol^20,21^, therefore Ni clusters are needed in the cracking process or hydrogenolysis reactions^22^. Together, these findings illustrate that the atomic-scale structure of the active sites in bimetallic catalysts has a critical effect on their chemical reactivity and selectivity. Therefore, in situ control of the structure of the active sites in bimetallic catalysts should offer a way to tune reaction networks and achieve efficient conversion to the desired products.

Catalysts are known to dynamically restructure under reactive atmospheres^23–29^. In metal alloy catalysts, the driving force for such changes is often derived from differences in the binding strength of an adsorbate to the different metallic components of the alloy. For example, Wu et al.^23^ reported that the CO oxidation process induced the reconstruction of CoPd bimetallic NPs, forming CoO_x_ species on the surface which promoted CO oxidation. McCue et al.^30^ reported on PdCu alloys for which CO treatment led to Pd atoms aggregating on the surface resulting in an improved activity for the acetylene hydrogenation reaction. Gao et al.^31^ reported the formation of contiguous Pd sites on an Au(100) surface at CO pressures higher than 0.1 Torr, which leads to an increased rate of CO_2_ formation. Considering these findings, understanding how the active sites in bimetallic catalysts reconfigure in response to changes in gas-phase composition is a critical step in unraveling intrinsic reaction mechanisms and ultimately controlling reaction pathways in situ.

While many bimetallic catalysts are difficult to model accurately, SAA catalysts with their simple structure in which the dopant atom substitutes a host metal atom, are more amenable to accurate modeling that includes the effect of restructuring^26,28,32–44^. Theoretical studies on SAAs by Darby et al.^42^ and Papanikolaou et al.^43,44^ predicted that the surface coverage of CO has an impact on the structure of the PGM ensembles present on the surface. Specifically, it was found that at different CO coverages (a variable that can be experimentally controlled), certain bimetallic catalysts exhibit stable dimers, trimers, or larger clusters in the surface. These theoretical studies, therefore, suggest a method for manipulating the structure of the active sites of a bimetallic catalyst in situ.

Here, we show the translation of these theoretical results to working catalysts and probe this effect experimentally using PdAu SAA nanoparticle catalysts supported on SiO_2_. Using in situ CO-DRIFTS, we monitor the structure of Pd ensembles and demonstrate that their structure can be reversibly tuned from isolated atoms to clusters by changing CO partial pressure as a function of temperature. These experimental observations are further rationalized using on-lattice Monte Carlo (MC) simulations parametrized by density functional theory (DFT), which show how the pressure of CO and temperature affect the geometry of the Pd ensembles. Furthermore, we demonstrate how in situ control of the active sites with CO can be used to transition the catalyst from a SAA phase that is selective to acetaldehyde and hydrogen to a Pd cluster phase which leads to the formation of CO, ethyl acetate, and CH_4_ as byproducts in the ethanol dehydrogenation (EDH) reaction. This should be a somewhat general method for in situ control of catalyst selectivity that can be predicted a priori by DFT and MC simulations.

## Results and discussion

### Predicted effect of CO on Pd segregation and aggregation

**Fig. 1:**
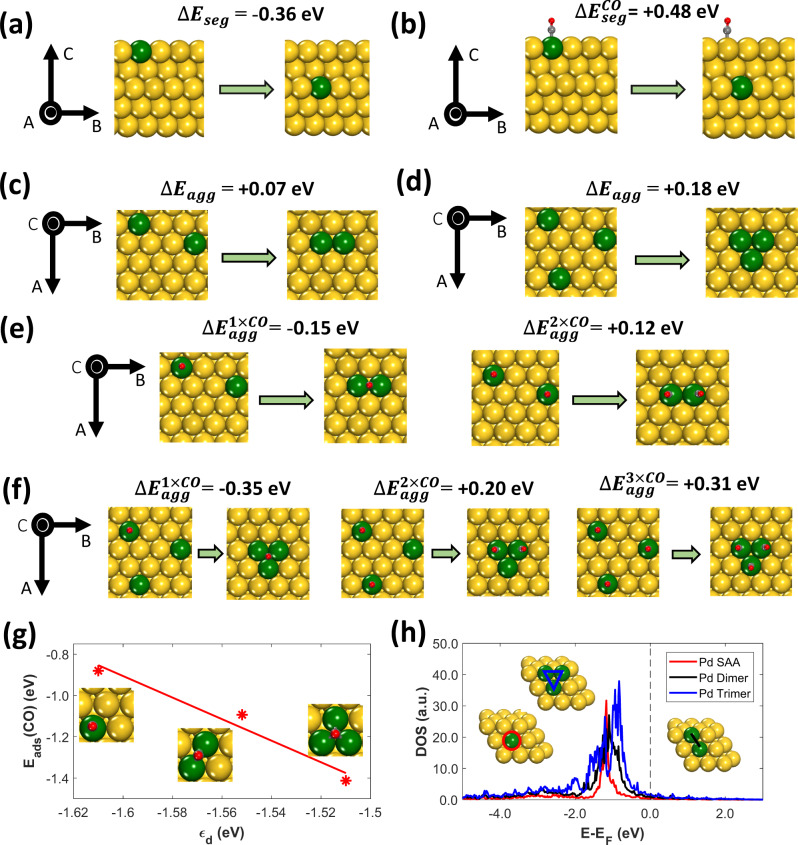
Predicted effect of CO on Pd segregation and aggregation. Initial and final states for (**a**) Pd segregation; **b** CO-induced Pd segregation; **c** Pd–Pd dimer aggregation; **d** Pd trimer aggregation; **e** CO-induced Pd–Pd dimer aggregation at different CO fractional coverages; and **f** CO-induced Pd trimer aggregation at different CO fractional coverages. Values of segregation or aggregation energies are shown on the top of each panel. The given segregation energies in panels (**a**) and (**b**) are per Pd atom; negative segregation energy (Δ*E*_seg_) values indicate an enthalpic preference for Pd to segregate into the bulk, while positive values indicate a preference for Pd segregation to the surface layer. The reported aggregation energies are given relative to the energy of a SAA; negative aggregation energy (Δ*E*_agg_) values imply a preference for Pd atoms to cluster into dimers, whereas when Δ*E*_agg_ > 0 there is a thermodynamic preference for the SAA phase (i.e., isolated Pd atoms in the Au surface). Panels (**g**) and (**h**) show a linear correlation between the CO adsorption energy (*E*_ads_(CO)) and the *d* band center of the *d* states of the Pd atoms that comprise the adsorption site, and *d* density of states (DOS) plots in which the DOS are projected onto the same atoms, respectively. Au, Pd, C, and O atoms are shown in yellow, green, gray and red, respectively. The Pd atoms onto which the *d* DOS are projected in panel (**h**) are highlighted in the colors of the corresponding DOS plots. In the schematics of the axes, the symbol ◉ denotes an arrow pointing from the page toward the reader.

A primary consideration for designing a working SAA catalyst is whether the catalytically active atoms will be stable in the surface layer of the nanoparticle and accessible to the reactants. Our theoretical calculations demonstrate that, under vacuum conditions, there is an enthalpic preference for Pd atoms to reside in the bulk of the Au host (Δ*E*_seg_ = −0.36 eV) as seen in Fig. 1a due to the higher surface energy of Pd compared to that of Au^45,46^. In contrast, in the presence of CO, the strong Pd–CO bond leads to Pd atoms exhibiting higher thermodynamic stability in the surface of the bimetallic alloy (${{\Delta }}E_{{\mathrm{seg}}}^{{\mathrm{CO}}}$ = +0.48 eV) (Fig. 1b), which drives CO-induced surface segregation.

In addition to surface segregation, our results suggest that CO is capable of inducing the surface aggregation of Pd atoms, thereby changing the size of the Pd ensembles at the catalyst surface^42–44^. Specifically, in the absence of CO, we compute positive aggregation energies for both Pd–Pd dimers (Δ*E*_agg_ = +0.07 eV) and Pd trimers (Δ*E*_agg_ = +0.18 eV). These values indicate that there is a thermodynamic preference for the Pd atoms to be isolated from one another in the PdAu surface (Fig. 1c, d)—(see “Methods” for the definition of the pertinent energy differences). Furthermore, our calculations show that the formation of the aforementioned Pd aggregates is favored at relatively low CO dopant fractional coverages (Fig. 1e, f)^43^. This is because the most stable adsorption sites for a single CO molecule are bridge or threefold hollow sites between adjacent dopant atoms leading to the formation of Pd dimers and trimers in the catalytic surface.

In contrast, at higher CO coverages, the CO adsorption geometries on dopant dimers or trimers are tilted away from one another. These repulsive CO–CO lateral interactions^42,43,47,48^ are expected to promote Pd dispersion back to single atoms (Fig. 1e, f). Therefore, it appears that CO can be used to stabilize the active site ensemble size in bimetallic alloys.

Regarding the binding of CO on the most stable adsorption site of the Pd ensembles (i.e., monomers, dimers, and trimers), we find that the CO-binding strength increases with increasing size of the Pd cluster (Fig. 1g); therefore, Pd dimers and trimers are expected to exhibit higher reactivity than isolated Pd atoms. Such a trend can be rationalized by the position of the *d* band center (*ε*_d_) of the *d* states of the atoms that comprise the adsorption site (Fig. 1g)^49^. We note that *ε*_d_ shifts to less negative values as the size of the Pd clusters increase and that *ε*_*d*_ scales linearly with *E*_ads_ (CO) (Fig. 1g). This observed shift of *ε*_d_ is the result of the *d* density of states (DOS) for dimers and trimers being closer to the Fermi level compared to Pd single atoms (Fig. 1h), thereby explaining not only the strong Pd cluster–adsorbate interactions, but also why the cluster samples have higher reactivity than the SAA, albeit at the cost of selectivity^17,50,51^.

### In situ IR of Pd clustering and redispersion induced by CO

**Fig. 2:**
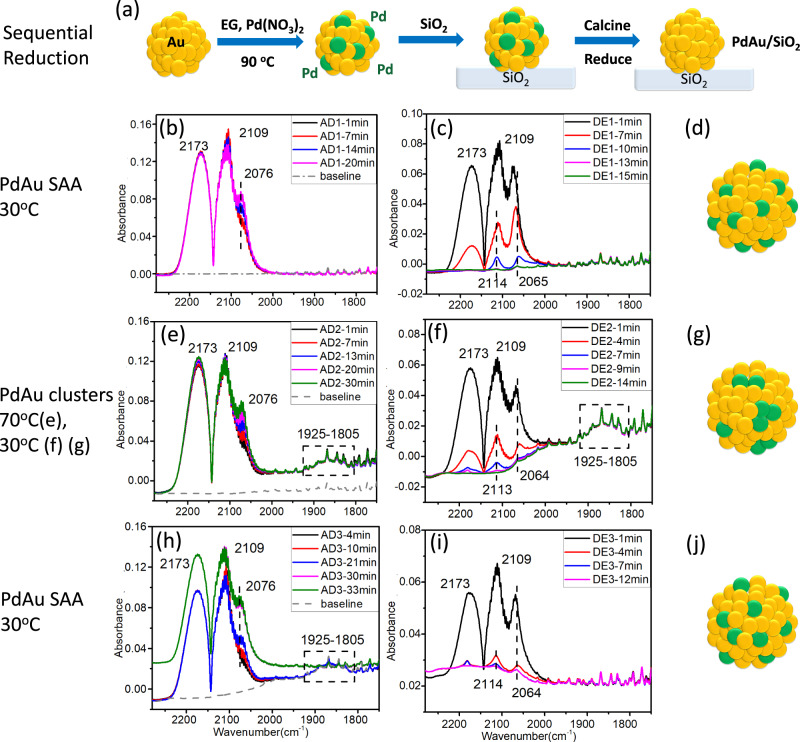
In situ IR data of Pd clustering and redispersion induced by CO. **a** Schematic illustration of PdAu/SiO_2_ synthesis in which the final reduction step leads to migration of Pd to the interior of the NPs. CO-DRIFTS results and proposed structures of a Pd_0.02_Au_0.98_ /SiO_2_ sample at 30 °C (**b**–**d**), 70 °C (**e**), 30 °C (**f**, **g**), 30 °C (**h**–**j**) during 10%CO/He adsorption (AD) (P_CO_ = 0.1 bar) and desorption (DE) process. Prior to the experiment, the sample was reduced at 200 °C in 5% H_2_/He for 1 h in situ. The labels in the upper right panels indicate the CO adsorption (AD), and desorption processes (DE) via He purge.

Inspired by these theoretical results, we synthesized and tested PdAu/SiO_2_ bimetallic catalysts. Pd_0.02_Au_0.98_ NPs with a Pd/Au atomic ratio of 1/49 and an average diameter of 7.6 ± 1.9 nm^52,53^ were prepared by the sequential reduction method and then supported on SiO_2_ (Fig. 2a and Supplementary Table S1). Our DFT calculations indicate that after the calcination and reduction process which removes all surface-bound species, the most thermodynamically stable state of Pd is in the bulk of the Au NPs (Fig. 1a). We used in situ CO-DRIFT spectroscopy to monitor the surface structure of the Pd_0.02_Au_0.98_/SiO_2_ catalysts and their dynamic response to CO in flow mode. In these experiments, CO induces restructuring of Pd ensembles in the surface of Pd_0.02_Au_0.98_ NPs, while simultaneously acting as the probe of the adsorption site types.

Figure 2 summarizes the synthesis and the IR characterization data, in which the as-synthesized Pd_0.02_Au_0.98_ /SiO_2_ catalysts are first reduced in H_2_ at 200 °C followed by CO adsorption (denoted as AD) and desorption (denoted as DE) in sequential temperature steps. To examine the effect of the H_2_ reduction treatment on Pd segregation to the surface, we computed the corresponding segregation energies. We find $\Delta E_{{\mathrm{seg}}}^H = - 0.12\,{\mathrm{eV}}$ and $\Delta E_{{\mathrm{seg}}}^{H_2} = - 0.15\,{\mathrm{eV}}$, which demonstrates the ineffectiveness of hydrogen (H and H_2_) at stabilizing Pd atoms originally present in the bulk in the surface^54^. Therefore, we expect that very little, if any, Pd segregation to the catalyst surface should occur prior to exposure to CO^54^. During CO adsorption at 30 °C (Fig. 2b), the two adsorption bands at 2173 cm^−1^ and 2109 cm^−1^ arise from the R and P branches of gaseous CO^55^. The shape of the peak at 2109 cm^−1^ is different from the usual P branch of gaseous CO peak, which results from its overlap with CO adsorbed on Au atop sites^56,57^. A shoulder at 2076 cm^−1^ forms and increases in intensity during the CO adsorption process (AD1-1min to AD1-20 min). This peak is attributed to CO-bound atop isolated Pd sites at high CO coverage^17,31,58^. The increase of this peak intensity with time indicates that after H_2_ reduction the Pd resides in the NP bulk and that CO induces the migration of Pd atoms to the surface because CO binds to Pd stronger than to Au^31,59^, in agreement with our DFT calculations. During the adsorption process, the intensity of the peak at 2109 cm^−1^ decreases, suggesting a decrease of Au atoms on the surface of the NP as they are replaced with Pd atoms. During the desorption process at 30 °C via helium purge (Fig. 2c), the gaseous CO signal decreases in intensity as gas-phase CO is flushed from the system, and two peaks at 2114 cm^−1^ and 2065 cm^−1^ are observed and assigned to atop CO adsorption on Au^56,57^ and isolated Pd sites^17,31,58^, respectively. There are no observable bands associated with bridge-bound CO, indicating that under these conditions, Pd is atomically dispersed in the surface of Au NPs (Fig. 2d). The shift of the atop CO peak on isolated Pd from 2076 cm^−1^ to 2065 cm^−1^ is attributed to decreased competition for metal d-electrons back-donated into the CO 2π* orbital or decreased dipole-dipole coupling interaction when the CO coverage decreased^56,60,61^.

After CO adsorption/desorption at 30 °C, we increased the temperature to 70 °C under helium flow followed by CO adsorption. After flowing CO for 1 min (Fig. 2e, AD2-1min), a broad feature in the region 1925–1805 cm^−1^ is observed, which is consistent with CO adsorbed on the bridge and threefold hollow sites, indicating the formation of Pd clusters^57,62^. The feature at 2076 cm^−1^ increases in intensity during the CO adsorption process (AD2-1min to AD2-30 min). This is because of the Pd atoms moving back into the Au NPs during the helium purge as there is no driving force to stabilize Pd in the surface unless CO is present and this gradual increase of the atop CO peak is consistent with the high barriers for the diffusion of Pd atoms in the bulk of Au nanoparticles to the surface layer. In contrast, the Pd clusters are formed from the coalescence of surface Pd atoms, and since metal carbonyl species on the surface typically encounter lower diffusion barriers, their aggregation toward these clusters is a fast process^63–65^. The system was then rapidly cooled down to 30 °C under helium flow to lock in the new structure of the catalyst. During the CO desorption process via helium purge (Fig. 2f), the broad peak at 1925–1805 cm^−1^ remains because CO binds more strongly to Pd clusters (Fig. 2e). Applying known extinction coefficients^66^ to our spectra in Fig. 2f—DE2-14min, which have a raw IR area ratio of linearly adsorbed CO to CO bound to more than one Pd of 1:25 leads to a corrected fraction of CO on isolated Pd sites to Pd clusters of ~1:1 in the Pd cluster sample.

We then repeated the CO adsorption/desorption process at 30 °C. During the initial 21-min CO adsorption process at 30 °C, the peaks at 2173 cm^−1^, 2109 cm^−1^, 2076 cm^−1^, and 1925–1805 cm^−1^ (Fig. 2h) remain the same as those of CO adsorption at 70 °C, corresponding to the presence of Pd clusters in the Au NP surface. The broad IR peak at 1925–1805 cm^−1^ disappeared after 30 min CO adsorption (Fig. 2h), AD3-30 min, and only the bands at 2114 cm^−1^ and 2064 cm^−1^ associated with atop-bound CO on Au and isolated Pd atoms remained after the desorption process (Fig. 2i). This indicates that the CO treatment at 30 °C re-disperses Pd clusters back to Pd atoms (Fig. 2j). The complete absence of small clusters suggests that these species are unstable when the surface coverage of CO is high enough at 30 °C. Therefore, our in situ CO-DRIFTS experiments demonstrate the dynamic structural changes of our PdAu catalysts in response to CO, from Pd single atoms to Pd clusters and redispersion back to single atoms, as a function of CO surface coverage which we control via temperature. While we find that the CO-induced rearrangement of Pd from atoms to clusters and then back to atoms is initially a reversible process, repeated cycling between phases leads to carbon buildup and eventual loss of complete reversibility of the process (see Supplementary Discussion and Supplementary Figs. S4 and  S5).

### Computational predictions of CO-induced ensemble tuning effects in PdAu

To further probe the mechanistic origin of the experimental CO-DRIFTS results, we performed on-lattice first-principles based MC simulations. These simulations account for important components of the free energy such as the configurational entropy contribution and the loss of translational and rotational degrees of freedom of CO upon adsorption.

In the presence of CO and at temperatures higher than 27 °C, the low-index (111) facet is expected to be the predominant one on Au NPs^67^. Therefore, our MC simulations focus on this latter surface and capture the restructuring of the top layer of the PdAu alloy when exposed to CO^43^. Indeed, the CO-DRIFTS results indicate that equilibrium with respect to the catalyst structure is reached within the presented experimental timescales (Fig. 2). For example, we note that the peak which is associated with CO adsorbed atop on Pd (2076 cm^−1^) exhibits very similar intensities in Fig. 2b, e, h after the longest time interval of CO exposure. On this basis, the goal of the MC simulation is to elucidate the most thermodynamically stable Pd configurations, while modeling of the dynamic restructuring of the PdAu catalyst that includes kinetic barriers for surface (restructuring) processes (e.g., atomic swaps, Pd segregation from the bulk to the surface layer) is part of ongoing work. Moreover, our DFT calculations indicate that the binding strength of CO on the Au(111) surface is only weakly perturbed by the presence of subsurface Pd clusters (~60 meV stronger CO binding on an Au(111) surface with a four atom Pd cluster in the subsurface layer than on a pure Au(111) slab—see Supplementary Fig. S9).

The interaction of CO with metal surfaces is known to be challenging to reproduce quantitatively with DFT^68–70^, and therefore we performed a sensitivity analysis to understand the effect of the binding strength of CO on the population of different Pd structures (i.e., Pd monomers, Pd–Pd dimers, and Pd trimers) (Supplementary Fig. S8). We find that DFT appears to overestimate the CO-binding energy as evidenced by (i) the absence of Pd clusters for the experimental conditions of 70 °C and P_CO_ = 0.1 bar, and (ii) the systematically lower vibrational frequencies for CO chemisorption on Pd atoms and aggregates in the Pd/Au(111) surface. In particular, the computed vibrational frequencies of CO on Pd atop, Pd–Pd bridge, and threefold sites are 2018 cm^−1^, 1859 cm^−1^, and 1755 cm^−1^, respectively, which are lower than the corresponding experimental values (i.e., 2076 cm^−1^, for Pd atop and between 1925 and 1805 cm^−1^ for Pd bridge and threefold), thereby indicating that the binding strength of CO is, in every case, overestimated by DFT^56,62^. In view of this, in our model we use a CO-binding energy which is lower than the DFT computed one by 0.2 eV; equivalently adsorption energy shifted by +0.2 eV, which is within the widely accepted error of DFT^71^. This value reproduces substantial populations of Pd clusters, in line with experimental data for the aforementioned conditions.

**Fig. 3:**
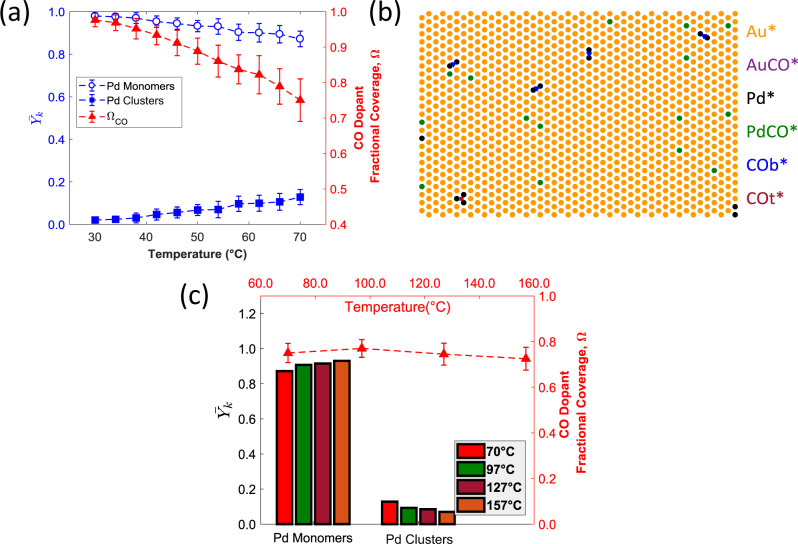
Computational investigations of CO-induced Pd clusters formation in Pd/Au. **a** Fractions of the different surface species ($\overline {Y_k}$) and CO dopant fractional coverage (Ω_CO_) at temperatures within the 30–70 °C range for *E*_ads_(CO) on Pd surface species that are shifted by +0.2 eV as compared to the DFT-predicted values; P_CO_ = 0.1 bar for all calculations. **b** Representative MC snapshot at 70 °C that shows that the majority of Pd aggregates are Pd–Pd dimers. Pd, Au, and Pd atoms with adsorbed CO are shown in black, yellow, and green, respectively. Bridge sites between Pd atoms, Au atoms covered by CO, and empty hollow sites are shown in light green, blue, and gray, respectively. Panel (**b**) shows only part of the MC lattice. (**c**) $\overline {Y_k}$ and Ω_CO_ at four different temperatures for Ω_CO_ ~0.75. The error bars in panels (**a**) and (**c**) are ±1 standard deviation.

We began the simulations by computing the fractions ($\overline {Y_k}$) of the different surface species (i.e., Pd monomers and Pd clusters) for P_CO_ = 0.1 bar and within the temperature range of 30–70 °C. Under these conditions, a 40 °C increase in temperature brings about a reduction of CO dopant fractional coverage (Ω_CO_) from ca. 0.99 to 0.75 (Fig. 3a). In turn, the decreased Ω_CO_ induces the surface aggregation of Pd atoms (Fig. 3a), thus elucidating the observed trends in the DRIFT spectra (Fig. 2). We also find that the majority of Pd clusters (ca. 77%) consist of Pd–Pd dimers (Supplementary Table S2 and Fig. 3b). The rest of the surface species contain mainly Pd trimers, while the fraction of Pd clusters with more than 3 Pd atoms is extremely small under the investigated conditions and such clusters rarely appear on the MC lattice (Supplementary Table S2).

Besides Ω_CO,_ there is also an important effect of temperature on the distribution of Pd surface species. To clarify this effect, we have carried out representative MC simulations for three additional temperatures (*T* = 97 °C, 127 °C, and 157 °C), while applying a P_CO_ that maintains Ω_CO_ ~0.75 (i.e., the computed Ω_CO_ value at P_CO_ = 0.1 bar and 70 °C in Fig. 3a–c). Our MC simulations indicate that the average fraction of Pd clusters diminishes at a higher temperature and that the SAA phase gains in thermodynamic stability (Fig. 3c). Thus, we postulate that at very high *T* (higher than 300 °C as shown in Supplementary Fig. S3), Pd aggregates will tend to disperse to single atoms. Indeed, one may expect that higher temperatures, which increase the entropy of the system, would favor the SAA phase owing to its higher disorder as compared to Pd clusters.

### Ethanol dehydrogenation on Pd_0.02_Au_0.98_/SiO_2_ after CO treatment

To understand the effect of the different Pd ensembles of the Pd_0.02_Au_0.98_/SiO_2_ samples on catalytic performance, ethanol dehydrogenation (EDH) was chosen as a probe reaction. SiO_2_ was used as an inert support and did not show any activity in the EDH reaction below 400 °C^20^. It has been reported that NiCu/SiO_2_ and NiAu/SiO_2_ SAAs catalyze the EDH reaction with 100% selectivity to acetaldehyde and H_2_, whereas catalysts consisting of Ni clusters break the C–C bond of ethanol, leading to enhanced activity but decreased selectivity, yielding CO and CH_4_^21,72^. PdCu/SiO_2_ SAA^20^ and atomically dispersed Pd/ZnO^73^ catalysts have also exhibited 100% selectivity to acetaldehyde and H_2_ in EDH, while Pd clusters lead to the decomposition of ethanol to CO. Evans et al.^74^ and Li et al.^75^ conducted computational studies of the EDH reaction mechanism on Pd/Au(111) surface alloys, demonstrating that Pd is the active site and the activity and selectivity of the reaction is heavily dependent on the type of Pd ensembles. These prior data suggest that PdAu/SiO_2_ catalysts in the SAA or cluster phases should exhibit different catalytic performance for the EDH reaction.

**Fig. 4:**
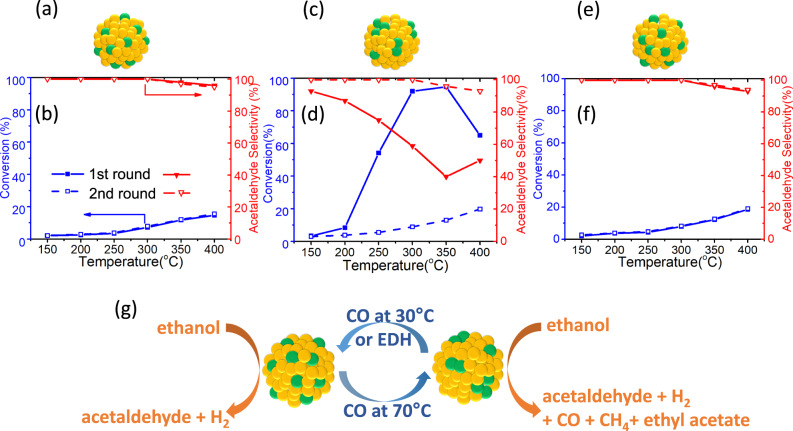
In situ control over a reaction pathway via CO treatment. Structure (**a**, **c**, **e**) and catalytic performance (**b**, **d**, **f**) of a Pd_0.02_Au_0.98_/SiO_2_ sample after (**a**, **b**) no CO treatment, (**c**, **d**) CO at 30 °C for 30 min then 70 °C for 30 min which causes Pd to form clusters, (**e**, **f**) CO treatment at 30 °C for 30 min, then 70 °C for 30 min, then 30 °C for 1 h which causes Pd to aggregate and then re-disperse back into atoms. **g** Schematic illustration showing how CO treatment can be used to change Pd from atoms to clusters and back, and the effect of these different active sites on reaction pathway. Ethanol dehydrogenation (EDH) reaction conditions: 300 mg catalyst, 2% ethanol in helium, total flow rate 12 mL/min, GHSV = 2400 mL/(h∙g_cat_). Each temperature was held for 2 h. The solid line shows the first-round EDH reaction from 150 °C to 400 °C; the dotted line is the second round reaction. Each point is the average of data obtained during 2 h.

We tested our Pd_0.02_Au_0.98_/SiO_2_ catalysts for the EDH reaction after different CO pretreatment conditions. As depicted in Fig. 4b, after H_2_ reduction at 200 °C, the reduced Pd_0.02_Au_0.98_/SiO_2_ SAA (Fig. 4a) exhibited low ethanol conversion (<20%) and 100% selectivity to acetaldehyde and H_2_ up to 300 °C. At temperatures above 350 °C, CO and methane is formed, which is characteristic of higher temperature Au chemistry (Supplementary Fig. S6)^76,77^. We then cooled the sample to 150 °C in ethanol flow, and a second reaction test was carried out while ramping the temperature from 150 °C to 400 °C. As can be seen in Fig. 4b, the catalytic performance during the second round was the same as that of the first round, which indicates that the SAA catalyst is stable under EDH reaction conditions. Compared to the performance of the Au/SiO_2_ catalyst (Supplementary Fig. S6), the addition of isolated Pd atoms to Au leads to an increase in the ethanol conversion while maintaining the high selectivity, illustrating the beneficial role of single Pd atoms, which is in line with literature reports^74,75^.

In Fig. 4d, we treated the reduced Pd_0.02_Au_0.98_/SiO_2_ SAA first under CO at 30 °C and then 70 °C (following the protocol used in the IR experiments) in order to drive the system towards the Pd cluster phase (Fig. 4c), as confirmed by the CO-DRIFTS results in Fig. 2f. The presence of Pd clusters in the catalyst leads to a dramatic increase in ethanol conversion at each temperature (up to 90% at 300 °C) compared to the SAA phase in Fig. 4b. Ethyl acetate was formed starting at 150 °C and decomposition products CO and CH_4_ were formed starting at 200 °C, leading to a significant decrease in acetaldehyde selectivity. The increased ethanol conversion and decreased selectivity observed in our study are in line with the reported behavior of Pd clusters in Pd/Au(111), PdCu, and Pd/ZnO catalysts for the EDH reaction^20,73–75^. We do not expect that CO formed during the reaction has any effect on the aggregation of Pd atoms because the maximum amount of CO formed in the product stream at the high-temperature extreme (350 °C) is only 0.6%, which is small compared to the 10% CO we used to control the Pd sites.

In the second round of tests, we cooled the sample to 150 °C under ethanol flow and ran the reaction tests up to 400 °C (Fig. 4d). We found under these conditions the original Pd cluster catalyst behaved like the pristine SAA catalyst, with low ethanol conversion (<20%) and 100% selectivity to acetaldehyde and hydrogen up to 300 °C. These results indicate that Pd clusters re-disperse back to Pd atoms under EDH reaction conditions. Therefore, we further checked the stability of the cluster Pd phase of the catalyst by carrying out longer performance tests. The performance of the Pd cluster catalyst was stable at 250 °C (Supplementary Fig. S3a). We noticed a significant decrease in ethanol conversion and increased acetaldehyde selectivity of the catalyst from 300 °C to 400 °C during a 60 h run and finally the same ethanol conversion and acetaldehyde selectivity as the pristine PdAu/SiO_2_ SAA catalyst is reached at 400 °C (Supplementary Fig. S3b). This result indicates that Pd clusters are not stable and revert to isolated Pd atoms at 400 °C under EDH reaction conditions. This conclusion is further supported by our MC simulations showing that elevated temperatures favor single Pd atoms as shown in Fig. 3c^43^.

In Fig. 4f, the reduced Pd_0.02_Au_0.98_/SiO_2_ SAA was treated with CO at 30 °C, 70 °C then 30 °C, 100% acetaldehyde selectivity was retrieved, and it behaved similarly to the pristine Pd_0.02_Au_0.98_/SiO_2_ SAA catalyst over two cycles. This result indicates that Pd aggregates formed at 70 °C re-disperse back to atoms after subsequent 30 °C CO treatment (Fig. 4e). The catalytic performance of a control Au/SiO_2_ catalyst sample did not show any changes after these different CO treatments. In addition, in situ ethanol-DRIFTS reveal the increased reactivity of Pd clusters present in the catalyst after the 70 °C CO treatment by the small number of ethoxy species left after the helium purge (Supplementary Fig. S1). This contrasts with the abundant ethoxy species left on the less reactive but more selective SAA catalyst (Supplementary Fig. S1), which is consistent with the reactor data.

These reaction results, coupled with the CO-DRIFTS and ethanol-DRIFTS data, demonstrate how CO-induced dynamic structural changes from a SAA to a Pd cluster structure can be induced and can be used to control reactivity and selectivity in the EDH reaction (Fig. 4g). We further demonstrate that the Pd clusters themselves slowly disperse back to Pd atoms at high temperature during the EDH reaction, hence the EDH reaction itself serves to stabilize the SAA structure which is most favorable for high reaction selectivity.

In summary, guided by DFT calculations we have demonstrated experimentally that the surface coverage of CO can be used to transition a PdAu alloy catalyst in situ between a SAA and a Pd cluster structure. Specifically, at a set CO pressure and lower temperature, the SAA is favored vs. at higher temperature, at which the lower CO surface coverage leads to the formation of Pd clusters. We further demonstrate that these two catalyst structures have distinctly different reactivity for the EDH reaction; the SAA phase is active for C–H activation but not C–C scission, yielding 100% selectivity to acetaldehyde and hydrogen, while Pd clusters decompose the ethanol leading to the formation of CO among other products. Furthermore, over time, and in the absence of CO, the EDH reaction itself re-disperses Pd clusters back to atoms with a concurrent change in the EDH reaction pathway. This in situ control of the atomic-scale surface structure in a bimetallic catalyst via CO treatment enables control over a reaction pathway. Our DFT calculations on a wide variety of other systems indicate that this approach is not unique to PdAu and should be generalizable to other bimetallic catalytic systems like NiAu and RhAu^42–44^.

## Methods

### Material synthesis

PdAu/SiO_2_ SAAs were prepared using sequential reduction, as previously reported^53^. Briefly, Au NPs were synthesized using 0.3 g HAuCl_4_·3H_2_O, 1.2 g poly(vinylpyrrolidone) (PVP, MW = 58,000), and 0.3 g NaHCO_3_ in 50 mL of ethylene glycol. The solution was heated to 90 °C and held for half an hour under nitrogen flow. The solution was then cooled to ambient temperature and the desired amount of Pd(NO_3_)_2_·xH_2_O was added. This solution was heated to 90 °C with vigorous stirring and held for 8 h. The resulting PdAu NPs were mixed with fumed silica (heat-treated in air at 650 °C for 3 h), dispersed in ethanol, and stirred overnight. The slurry carrying the supported NPs was washed and dried in a vacuum for 24 h before being calcined in air at 350 °C for 4 h resulting in Pd_0.02_Au_0.98_/SiO_2_ catalysts. Au/SiO_2_ catalysts were synthesized using the same procedure without the addition of Pd(NO_3_)_2_·xH_2_O.

### Characterization

PdAu/SiO_2_ samples were dissolved in concentrated HCl and H_2_O_2_ to prepare them for inductively coupled plasma atomic emission spectroscopy (ICP-AES) with a Leeman Labs PS1000 instrument.

Diffuse reflectance infrared Fourier transform spectroscopy (DRIFTS) was performed on a Thermo Scientific Nicolet iS50 FTIR spectrometer using a Praying Mantis high-temperature reaction chamber with a gas inlet and vent to allow the flow of pretreatment and reactant gases. Liquid nitrogen cooled MCT detector was used, and spectra acquired from 120 scans at 4 cm^−1^ resolution.

For in situ CO-DRIFTS, prior to spectra collection 10 mg PdAu/SiO_2_ samples were reduced in 10% H_2_/He at 200 °C for 1 h, then purged with He and cooled down to 70 °C and 30 °C to acquire background spectra (Supplementary Fig. S2). 10% CO/He was introduced into the cell to allow the adsorption of CO (noted as AD), then the system was quickly cooled down to 30 °C to freeze the structure of the sample and flushed with helium to remove any gas-phase CO (noted as DE), which has vibrational bands that overlap with peaks associated with surface-adsorbed CO. During the process, time-dependent spectra were recorded. The baseline was collected under He before any CO was introduced.

For in situ ethanol-DRIFTS, 10 mg of samples were pretreated in situ at 200 °C with a 10% H_2_/He mixture for 1 h prior to purging with helium. The background spectra were collected under helium at 200 °C and 130 °C (Supplementary Fig. S2), respectively. Then some catalysts underwent CO treatment and others that did not served as controls. Catalysts were then saturated with vaporized ethanol carried by a helium flow at 130 °C or 200 °C (noted as AD). When the IR spectra did not change with time, meaning the saturation was complete, a helium purge was conducted to remove gaseous ethanol and characterize the sample surface (noted as DE).

Temperature-programmed oxidation (TPO) was carried out in a Micromeritics AutoChem II 2920 instrument equipped with a Blazer ThermoStar quadrupole mass spectrometer. Fragments of *m/z* = 32, 44, and 18, corresponding to O_2_, CO_2_, and H_2_O were monitored using the mass spectrometer. The test was performed on the 100 mg as-synthesized Pd_0.02_Au_0.98_/SiO_2_, Au/SiO_2_ catalysts, and SiO_2_ support, which was first calcined in 3%O_2_/Ar at 350 °C for 1 h, then reduced under 10% H_2_/Ar at 200 °C for 1 h. The temperature was then lowered to 35 °C, and some samples were exposed to the 10% CO/He treatment at 35 °C and/or 70 °C, while some were not as indicated in Supplementary Fig. S4b. Then a He purge at 300 °C for 1 h was conducted to remove both surface-adsorbed CO and gaseous CO. Finally, the sample underwent TPO with 3% O_2_/Ar from 40 °C to 600 °C at a rate of 2 °C/min. The products (H_2_O and CO_2_) were monitored using a quadrupole mass spectrometer.

### Catalytic activity measurements

The ethanol dehydrogenation activity of the PdAu/SiO_2_ samples was evaluated in a fixed-bed flow reactor at atmospheric pressure. Typically, a 300 mg of sample mixed with 700 mg of silica sand was loaded into a quartz reactor tube packed in between two quartz wool plugs. The reactor was heated in a furnace equipped with a temperature controller. The temperature of the catalyst was measured with a K-type thermocouple in contact with the top of the catalyst bed. Before reaction, the catalyst was reduced with 10% H_2_/He at 200 °C for 2 h. Ethanol was injected into a flow of pure He by a syringe pump and vaporized in the heated gas line before entering the reactor. The ethanol concentration was 2wt.% and the total flow rate was 12 mL/min, corresponding to a gas hourly space velocity (GHSV) of 2400 mL/(h∙g_cat_). The effluent products were monitored online by two gas chromatographers in series. The first gas chromatograph (HP 5890) with an HPLQ capillary column (Hewlett-Packard Company, 30 m × 0.32 mm × 0.50 μm) was equipped with a thermal conductivity detector (TCD) to monitor ethanol, acetaldehyde, ethyl acetate, or other oxygenates. Then the gas went through the condenser and the effluent was monitored via a SRI 331 gas chromatograph equipped with a Carbosphere 80/100 packed column and TCD detector to monitor CO, CH_4_, and CO_2_.

### Density functional theory calculations

DFT calculations were carried out using the Vienna Ab initio Simulation Package (VASP)^78,79^. All calculations were performed within the generalized gradient approximation (GGA), and the revised Perdew−Burke−Ernzerhof (RPBE) functional was employed to treat exchange and correlation effects^80,81^. The interactions between the frozen core and valence electrons were described by the projector augmented wave (PAW) method^82^, and the kinetic energy cutoff for the plane-wave basis set was 400 eV. The chemisorption of CO on PdAu alloy surfaces was explored using a five-layer *p*(3 × 3) slab, and the Brillouin zone was sampled with a 13 × 13 × 1 Monkhorst–Pack k-point mesh. During geometry optimization, the two bottom Au layers were kept fixed at the RPBE-calculated Au lattice constant (i.e., 4.22 Å)^83^, while the three top layers and any adsorbate species were allowed to fully relax. Electronic self-consistency was achieved up to a tolerance of 10^−7^ eV/Å, and the force convergence criterion for the ions that were free to move was 0.01 eV/Å. Vibrational analyses were performed under the harmonic approximation with a finite-difference displacement of 0.02 Å.

The adsorption energy of *m* CO molecules was computed using the following expression:

1 $$E_{{\mathrm{ads}}}\left( {m \times {\mathrm{CO}}} \right) = E_{{\mathrm{tot}}}^{m \times {\mathrm{CO}} + {\mathrm{slab}}} - E_{{\mathrm{tot}}}^{{\mathrm{slab}}} - m \times E_{{\mathrm{tot}}}^{{\mathrm{CO}}_{\left( g \right)}},$$

where $E_{{\mathrm{tot}}}^{m\times{\mathrm{CO}} + {\mathrm{slab}}}$ is the DFT total energy of m chemisorbed CO molecules on the PdAu surface, $E_{{\mathrm{tot}}}^{{\mathrm{slab}}}$ is the DFT total energy of the clean slab, and $E_{{\mathrm{tot}}}^{{\mathrm{CO}}_{(g)}}$ is the DFT total energy of a CO molecule in the gas phase. According to this definition, the more negative the *E*_ads_(*m* × CO) value, the stronger the binding of CO on the PdAu surface.

The surface segregation energy (Δ*E*_seg_) indicates the thermodynamic preference of Pd atoms to reside in the bulk of the Au matrix or in the surface layer. Similar to our previous works, the segregation energies in the absence (Δ*E*_seg_) and in the presence of CO (Δ$E_{{\mathrm{seg}}}^{{\mathrm{CO}}}$) were defined by Eq. (2) and Eq. (3), respectively.

2 $$\Delta E_{{\mathrm{seg}}} = E_{{\mathrm{tot}},{\mathrm{Bulk}}} - E_{{\mathrm{tot}},{\mathrm{SAA}}},$$

3 $$\Delta E_{{\mathrm{seg}}}^{{\mathrm{CO}}} = \Delta E_{{\mathrm{seg}}} + \left\{ {E_{{\mathrm{ads}}}^{{\mathrm{host}}}\left( {{\mathrm{CO}}} \right) - E_{{\mathrm{ads}}}^{{\mathrm{SAA}}}\left( {{\mathrm{CO}}} \right)} \right\},$$

where $E_{{\mathrm{tot}},{\mathrm{Bulk}}}$ is the DFT total energy of a Pd atom placed in the third layer of the slab; $E_{{\mathrm{tot}},{\mathrm{SAA}}}$ is the DFT total energy of the Au slab with a Pd atom in the surface layer; $E_{{\mathrm{ads}}}^{{\mathrm{host}}}\left( {{\mathrm{CO}}} \right)$ is the adsorption energy of a CO molecule on the top site of an Au atom and $E_{{\mathrm{ads}}}^{{\mathrm{SAA}}}\left( {{\mathrm{CO}}} \right)$ is the adsorption energy of a CO molecule on the SAA surface. Negative values of the segregation energy suggest a preference of Pd to move from the surface into the bulk of Au, while positive values imply the opposite.

Moreover, the thermodynamic stability of Pd aggregates on the surface layer of the PdAu alloy (e.g., Pd–Pd dimers) can be compared to the stability of single Pd atoms thereon by means of the aggregation energy (Δ*E*_agg_). The latter energy, under vacuum conditions, is given by Eq. (4) while in the presence of chemisorbed CO is given by Eq. (5).

4 $$\Delta E_{{\mathrm{agg}}} = E_{{\mathrm{tot}}}\left( n \right) + \left( {n - 1} \right) \times E_{{\mathrm{tot}}}\left( {{\mathrm{host}}} \right) - n \times E_{{\mathrm{tot}}}\left( {{\mathrm{SAA}}} \right),$$

5 $$\Delta E_{{\mathrm{agg}}}^{m \times {\mathrm{CO}}}\left( n \right) = \Delta E_{{\mathrm{agg}}}\left( n \right) - \left\{ {m \times E_{{\mathrm{ads}}}^{{\mathrm{SAA}}}\left( {{\mathrm{CO}}} \right) - E_{{\mathrm{ads}}}^{n - {\mathrm{mer}}}\left( {m \times {\mathrm{CO}}} \right)} \right\},$$

where $E_{{\mathrm{tot}}}\left( n \right)$, $E_{{\mathrm{tot}}}\left( {{\mathrm{host}}} \right)$, and $E_{{\mathrm{tot}}}\left( {{\mathrm{SAA}}} \right)$ are the DFT total energies of an alloy with an aggregate of *n* Pd atoms, the pure host material, and the SAA, respectively; $E_{{\mathrm{ads}}}^{n - {\mathrm{mer}}}\left( {{\mathrm{CO}}} \right)$ and $E_{{\mathrm{ads}}}^{{\mathrm{SAA}}}\left( {{\mathrm{CO}}} \right)$ are the computed adsorption energies of the most stable configuration of *m* CO molecules on a Pd aggregate with *n* atoms, and of a CO molecule on the top dopant site of the Pd monomer, respectively. Finally, based on the definition of Δ*E*_*agg*_ in Eq. (4) and Eq. (5), negative values of Δ*E*_agg_ suggest that the formation of aggregates of Pd atoms is thermodynamically favored over the formation of isolated Pd atoms, while the opposite is true for Δ*E*_agg_ > 0.

### Monte Carlo simulations

On-lattice MC simulations were performed using *Zacros* (version 2.0)^84–86^, a software application that employs the graph-theoretical framework of Stamatakis et al.^87–91^. This framework is equally effective in performing equilibrium (Markov Chain MC (MCMC)) or dynamic (kinetic MC (KMC)) simulations of physicochemical phenomena on surfaces. For a surface represented by a lattice, the accessible (micro)states within the state space **Ω** capture all possible arrangements of adsorbates thereon, and the MC method simulates state-to-state transitions within **Ω**. Supplementary Figs. S11 and  S12 summarize the possible transitions in our simulations, which include adsorption, desorption and diffusion of CO, and Pt–Au atomic swaps. The probability of finding the system in state *i,* P_*i*_(**t**), is given by the Markovian Master Equation:

6 $$\frac{{{\mathrm{d}}P_i\left( t \right)}}{{{\mathrm{d}}t}} = - \mathop {\sum}\limits_{i \ne j} {k_{ij}P_i\left( t \right) + \mathop {\sum }\limits_{i \ne j} k_{ji}P_j\left( t \right)}$$

where *k*_*ij*_ and *k*_*ji*_ are the rate constants of surface processes that bring the system from state *i* to state *j* and vice versa. Realizations (sample trajectories) of the stochastic process captured by the Master equation are thus obtained by the MC method, thereby enabling the calculation of observables such as the CO coverage or the size distribution of Pd surface clusters.

For all simulations, the Pd loading of the PdAu surface was 2% atomic ratio on a lattice with a total number of 2500 metal atoms. The total number of sites was 14,700, including top and hollow (i.e., bridge or threefold) site types (Supplementary Fig. S10). The threefold sites represented both fcc and hcp hollow sites. The adsorption of CO on mixed metal sites (i.e., bridge sites between an Au and a Pd atom and threefold sites surrounded by both Pd ad Au sites) is not stable. Accordingly, these configurations are assigned with a large energetic contribution to the total energy, thereby assuring their instability in our MC simulations^43^. To replicate the CO-DRIFTS conditions, our MC simulations were performed at P_CO_ = 0.1 bar, and at various temperatures within the range of 30–70 °C. The CO dopant fractional coverage was calculated as follows:

7 $$\Omega _{{\mathrm{CO}}} = \frac{{N_{{\mathrm{CO}} \ast }}}{{N_{{\mathrm{Pd}}}}},$$

where *N*_CO_* is the number of CO molecules adsorbed on Pd sites, and *N*_Pd_ is the total number of Pd atoms on the lattice.

The lattice energetics were predicted by a cluster expansion (CE), which was fitted based on a DFT dataset^43^. This CE was assessed by the leave-one-out cross-validation score, which was found equal to 3.0 meV/site^43^. For the interested reader, more information on the fitting and performance of the CE can be found elsewhere^43^. During the simulation, several state-to-state steps could happen, including atomic swaps between Au and Pd atoms, CO diffusion from site-to-site, and CO adsorption/desorption. The full list of the microscopic processes that can take place on the MC lattice is presented in the Supporting Information. Our calculations aimed at sampling the surface configurations that show the highest thermodynamic stability and did not account for kinetic effects related to Au/Pd atomic swaps. The average fractions of surface Pd species (i.e., Pd monomers and Pd aggregates) were computed from Eq. (8) after reaching stationary conditions. For the latter, we checked that the CO coverage and the average fractions of the surface Pd species ($\bar Y_k$) remain approximately constant with respect to the simulation time.

8 $$\bar Y_k = \frac{1}{{N_{{\mathrm{MC}},{\mathrm{conf}}}}}\mathop {\sum}\limits_{i = 1}^{N_{{\mathrm{MC}},{\mathrm{conf}}}} {Y_{k,i} = \frac{1}{{N_{{\mathrm{MC}},{\mathrm{conf}}}}}} \mathop {\sum}\limits_{i = 1}^{N_{{\mathrm{MC}},{\mathrm{conf}}}} {\left( {\frac{{N_{k,i}}}{{N_{{\mathrm{tot}},i}}}} \right),k \in \left\{ {{\mathrm{Pdmonomers}},{\mathrm{Pdaggregates}}} \right\},}$$

where $\bar Y_{k,i}$ is the fraction of dopant species *k* in snapshot *i, N*_MC,conf_ is the number of lattice snapshots taken under stationary conditions, *N*_*k,i*_ is the number of dopant species *k* in snapshot *i* and *N*_tot*,i*_ is the total number of species detected in snapshot *i*.

## Supplementary information

Supplementary Information

## Data Availability

The data that support the plots within this paper and other findings of this study are available from the corresponding author upon reasonable request.

## Code availability

The codes that support the findings of this study are available from the corresponding author upon reasonable request.
