# Supplementary material for: Directing reaction pathways via in situ control of active site geometries in PdAu single-atom alloy catalysts
Source: Nat Commun. 2021 Mar 9;12:1549. doi: 10.1038/s41467-021-21555-z (PMC7943817; doi:10.1038/s41467-021-21555-z)
Supplement: Supplementary file 1 — Supplementary Information [file 41467_2021_21555_MOESM1_ESM.pdf]

Supplementary Information

Directing Reaction Pathways via in situ Control of Active Site Geometries in PdAu Single-Atom Alloy Catalysts

Mengyao Ouyang[1], Konstantinos G. Papanikolaou[2], Alexey Boubnov[3], Adam S. Hoffman[3], Georgios Giannakakis[1], Simon R. Bare[3], Michail Stamatakis[2], Maria Flytzani-Stephanopoulos[1], E. Charles H. Sykes[4]*

[1] Department of Chemical and Biological Engineering, Tufts University, Medford, MA, 02155, USA
[2] Thomas Young Centre and Department of Chemical Engineering, University College London, Roberts Building, Torrington Place, London WC1E 7JE, UK
[3] Stanford Synchrotron Radiation Light Source, SLAC National Accelerator Laboratory, Menlo Park, CA, 94025, USA
[4] Department of Chemistry, Tufts University, 62 Talbot Avenue, Medford, MA, 02155, United States
* Corresponding Author: charles.sykes@tufts.edu

Supplementary Discussion

In situ ethanol-DRIFTS and background spectra

To further compare the catalytic performance of the $Pd_{0.02}Au_{0.98}/SiO_2$ catalysts after various CO treatments, a study of the ethanol dehydrogenation (EDH) reaction was carried out and monitored by infrared spectroscopy (Supplementary Figure 1) under reaction conditions. During ethanol flow, molecular ethanol adsorbs on the catalyst surface and some forms the ethoxy intermediate as indicated by the DRIFTS peaks at 2983 cm^{-1} ($\upsilon_{as}(CH_3)$), 2938 cm^{-1} ($\upsilon_{as}(CH_2)$), 2901 cm^{-1} ($\upsilon_{s}(CH_3)$), 2884 cm^{-1} ($\upsilon_{s}(CH_2)$) [1,2]. After desorption induced by a He purge, a decrease of these band intensities indicated the desorption/reaction of the chemisorbed ethanol and surface ethoxy groups. The features at 1770 cm^{-1} and 1730 cm^{-1} are associated with the C=O stretching vibration of acetaldehyde [2,3]. The broad peak at 1860 cm^{-1} is ascribed to CO adsorbed in bridge and 3-fold hollow sites on Pd clusters [4,5], but this peak is small making it difficult to compare between the three catalysts due to the small amount of CO produced by Pd cluster catalysts. When comparing the intensity of ethoxy species after the desorption process, the ethoxy species IR peaks almost disappeared in Supplementary Figure 1 (b), indicating the probable decomposition of ethoxy species on this catalyst. This result is consistent with the existence of Pd clusters after the 70°C CO treatment, which leads to its high ethanol conversion in the EDH reaction test (Figure 4 (d)). On the other hand, the intensity of ethoxy species in Supplementary Figure 1 (a) and (c) are high, consistent with the fact that these catalysts have a Pd present as isolated atoms with lower reactivity. The in situ ethanol-DRIFTS results agree with the DFT, CO-DRIFTS and reaction test results indicating that the Pd structure can be tuned from single atom Pd to Pd clusters, and back to single atoms by changing the CO surface coverage with temperature.

Stability tests

In order to ascertain why the catalytic performance of the Pd cluster catalysts in second round tests are identical to the pristine SAA sample in Figure 4 (d), we conducted stability tests on the $Pd_{0.02}Au_{0.98}/SiO_2$ sample after the 70°C CO treatment, which induces Pd cluster formation. We first performed the EDH reaction at 150°C and 200°C for 2 h, then the stability test at 250°C for 20 h. We found no obvious change in the catalytic performance over 20 h at 250°C (Supplementary Figure 3 (a)), which was identical to the Pd cluster catalysts in Figure 4 (d). Upon increasing the temperature to 300°C, 350°C and 400°C and running the reaction at each temperature for 2 h, the activity of the catalyst decreased, and selectivity increased. Then, when we cooled the temperature down to 150°C under ethanol flow and ran the reaction from 150°C to 400°C again the performance of the catalyst returned to the same as the pristine $PdAu/SiO_2$ SAA as shown in Figure 4 (d). These results indicate that the dramatic structural change of Pd clusters occurs at temperatures higher than 250°C.

We then took a new batch of PdAu catalyst and applied the 70°C CO treatment to induce the Pd cluster formation and conducted stability tests at 300°C, 350°C and 400°C for 20 h at each temperature (Supplementary Figure 3 (b)). At 150°C, 200°C and 250°C, the performance of the catalyst was the same as the Pd cluster catalyst. Over the course of 20 h at 300°C, ethanol conversion decreased, and acetaldehyde selectivity increased. At 350°C, the change of the catalytic performance was even more obvious and reached a steady state in 20 h. At 400 °C the ethanol conversion was much lower than that of Pd cluster catalyst in Figure 4 (d), and eventually reached the same conversion and selectivity we observed for Pd SAA catalysts. These results indicate that Pd clusters are stable at 250°C, and re-dispersion of Pd clusters back to atoms occurs at 300°C which are both enthalpically and entropically favored at this temperature as described in theory section Figure 3 (c).

Cycling between single Pd atoms and clusters

The rearrangement of Pd from atoms to clusters and then back to atoms as a function of CO treatment should be a reversible process, so we performed more CO treatments on $Pd_{0.02}Au_{0.98}$ /SiO_2 catalysts in the DRIFTS cell. After CO treatment at 30°C, 70°C and 30°C as shown in Figure 2, we increased the temperature again to induce the formation of Pd clusters. No bridge or 3-fold hollow CO adsorption site was observed until 160°C as shown in Supplementary Figure 4 (a). At 160°C the formation of a broad feature in the same region 1925-1805 cm^{-1} appeared and is attributed to CO adsorbed in bridge and 3-fold hollow sites, indicating the formation of Pd clusters [4,5]. The peak at 2076 cm^{-1} is smaller than that at 30°C or 70°C, which results from less Pd in the surface at high temperature [6]. It is expected that at high temperature Pd atoms go back into the bulk, since Pd–CO binding is not sufficiently strong to stabilize Pd in the surface. Then we decreased the temperature to 30°C and during the CO adsorption process, this broad 1925-1805 cm^{-1} feature persisted for 2 h, suggesting that Pd clusters remain and do not re-disperse into atoms. This irreversibility is linked to carbon deposition on Pd clusters from CO decomposition as discussed below [6].

To verify this hypothesis about carbon deposition we conducted TPO on the $Pd_{0.02}Au_{0.98}$ /SiO_2, Au/SiO_2, SiO_2 samples after various CO or He treatments. As shown in Supplementary Figure 4 (b), we did not observe CO_2 formation on SiO_2 and pristine $Pd_{0.02}Au_{0.98}$ /SiO_2 (black and red line). After CO treatment at 35°C, there was a similar amount of CO_2 formation on the $Pd_{0.02}Au_{0.98}$ /SiO_2 SAA and the Au/SiO_2 catalysts (green and blue line), indicating that most of the carbon deposition occurs on Au instead of Pd atoms. The CO_2 signal shifted to lower temperature as the addition of Pd to Au/SiO_2 facilitates the oxidation of carbon [7]. After 70°C CO treatment, the amount of CO_2 formed on the $Pd_{0.02}Au_{0.98}$ /SiO_2 sample (pink line) was larger than that after a 35°C CO treatment, indicating that carbon deposition indeed occurs on Pd clusters. The carbon built up on the Pd clusters must therefore prevent the complete re-dispersion of Pd clusters back to atoms as C atoms covering the Pd clusters prevent CO from reaching the Pd [8].

To limit the effect of carbon deposition on Pd clusters and hence control the catalyst selectivity for the EDH reaction, H_2 and O_2 redox treatments were performed to remove surface carbon and surface reaction intermediates. After O_2 treatment Pd is partially oxidized and is enriched on the surface of Au [9-11]. After H_2 treatment the Pd oxide is reduced and Pd is atomically dispersed in Au [12] as demonstrated by our DRIFTS data. The pre-treatments for the catalytic tests are shown in Supplementary Figure 5 (a). In the first EDH reaction test (Supplementary Figure 5(b)), the reduced $Pd_{0.02}Au_{0.98}$ /SiO_2 SAA catalyst exhibited low ethanol conversion and 100% acetaldehyde selectivity consistent with the SAA phase. Then redox treatment was performed to remove surface adsorbates, followed by the 70°C CO treatment to induce Pd clustering. In the second EDH test, the ethanol conversion increased, and acetaldehyde selectivity decreased (Supplementary Figure 5 (c)) due to the presence of Pd clusters. After that, redox and CO treatment at 70°C and 30°C were conducted to re-disperse Pd clusters to atoms. However, in the third EDH reaction test (Supplementary Figure 5 (d)), the performance of the $Pd_{0.02}Au_{0.98}$ /SiO_2 sample did not completely return to the same performance as the SAA catalyst in the first test. However, ethanol conversion decreased, and acetaldehyde selectivity increased compared to the Pd cluster catalyst in the second test. This result indicates that Pd clusters do not completely re-disperse back to the single atoms before the third EDH test. This may result from the leftover carbon or increased size of the Au NPs due to the high temperature redox treatment.

Sensitivity analysis on CO binding strength

Supplementary Figure 8 shows the result of the sensitivity analysis of the CO binding strength over the PdAu alloy surface. The result reveals that Pd clusters are formed for a uniform reduction of the CO binding strength (i.e. less negative $E_{ads}(CO)$ on all types of sites) larger than 0.15 eV (area shaded in green in Supplementary Figure 8) In particular, a uniform shift of +0.20 eV in the DFT-predicted adsorption energy of CO ($E_{ads}^{DFT}(CO)$) gives rise to an average fraction of Pd aggregates ($\overline{Y_{Aggregates}}$) of ca. 0.13 (Supplementary Figure 8). Importantly, we note that deviations of 0.1 - 0.2 eV between DFT and experiment are common when using GGA functionals for modelling the adsorption of species on transition metal surfaces [13]. Furthermore, another source for the discrepancy between CO-DRIFTS and the ab initio MC simulations may be the presence of other facets, besides the low-index (111), on the synthesized PdAu NPs [14]; this is the so-called materials gap between surface science/theory and catalysis. The dopant aggregation on these other facets may differ, to some extent, from that on the (111) surface [15]. On this basis, we simulate the effect of a temperature rise from 30 to 70°C on the structure of the PdAu surfaces after uniformly shifting $E_{ads}(CO)$ on all the Pd aggregates by +0.2 eV (see the main text).

The effect of subsurface Pd on the CO binding strength over Au(111) is studied by replacing Au atoms by Pd atoms in the subsurface layer of our 3 x 3 DFT slab. The result indicates the presence of Pd clusters of even four atoms in the subsurface layer brings about a small change to the CO binding energy (ca. 60 meV – Supplementary Figure 9).

Microscopic processes included in the Monte Carlo reaction mechanism

Supplementary Figure 10 shows a representation of the MC lattice for the $Pd_{0.02}Au_{0.98}$ surface. The microscopic processes included in the reaction mechanism of our Monte Carlo simulations are shown in Supplementary Figure 11 and Supplementary Figure 12. Supplementary Figure 11 shows the atomic swaps and CO diffusions, whereas Supplementary Figure 12 displays the CO adsorptions/desorption.

Pd surface species analysis

Supplementary Table 2 tabulates the average fraction of the Pd surface species at different temperatures within the investigated temperature range. The last column is the sum of the Pd clusters (i.e. Pd-Pd dimers, trimers and multimers). The Pd multimers are defined as surface species with more than three Pd atoms on neighbouring positions, and as seen the average fraction remains at zero for almost all the examined temperatures. By contrast, Pd-Pd dimers are always the predominant species in the clusters. At 70°C the total average fractions of aggregate species become significant ($\overline{Y_{Aggregates,total}}$ = 0.13).

Calculation of surface segregation and aggregation energies

The segregation and aggregation energies are calculated from DFT-computed energies and using eq. (2) - (5) – (see the Computational Methods section). Supplementary Figure 13 shows how these segregation and aggregation energies, in the presence and absence of CO are calculated.

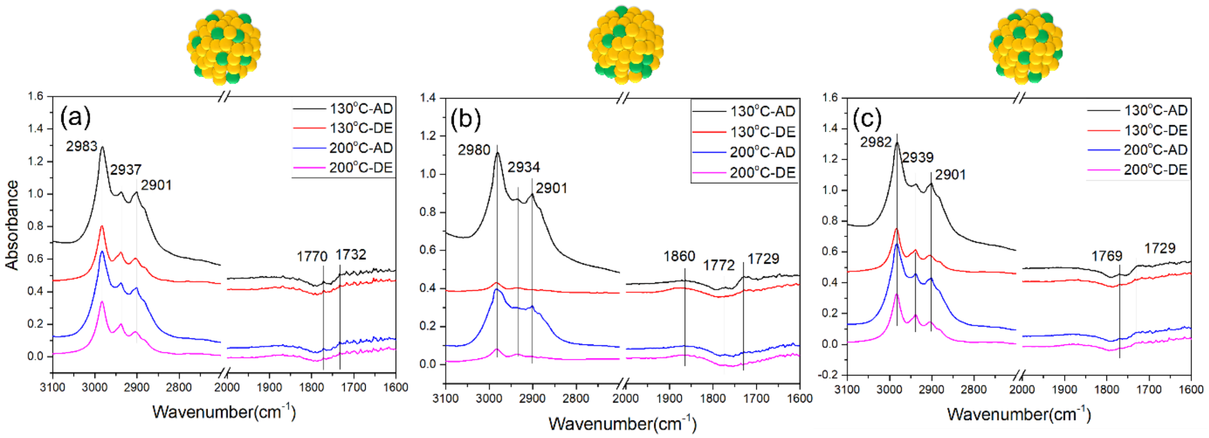

Supplementary Figure 1. In situ ethanol-DRIFT spectra of $Pd_{0.02}Au_{0.98}$ /SiO_2. The samples first underwent (a) no CO treatment, (b) CO treatment at 30°C for 30 min then at 70°C for 30 min, (c) CO treatment at 30°C for 30 min, then 70°C for 30 min, then 30°C for 1 h. The samples were then treated by He purge and sequentially under: (black line) CH_3CH_2OH-saturated He flow at 130°C, (red line) pure He at 130°C, (blue line) CH_3CH_2OH-saturated He flow at 200°C, and (magenta line) pure He at 200°C.

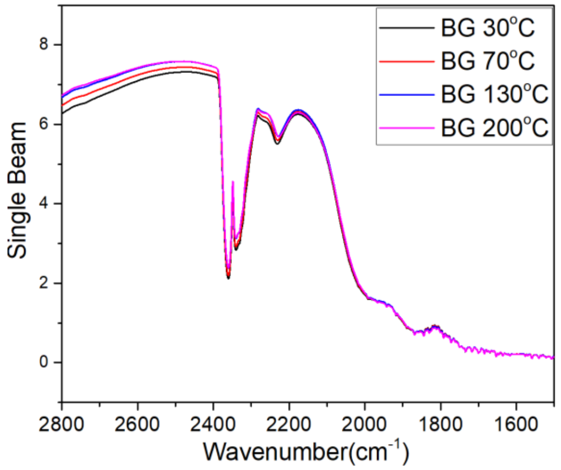

Supplementary Figure 2. DRIFT backgrounds of reduced $Pd_{0.02}Au_{0.98}$/SiO_2 sample under helium at different temperatures.

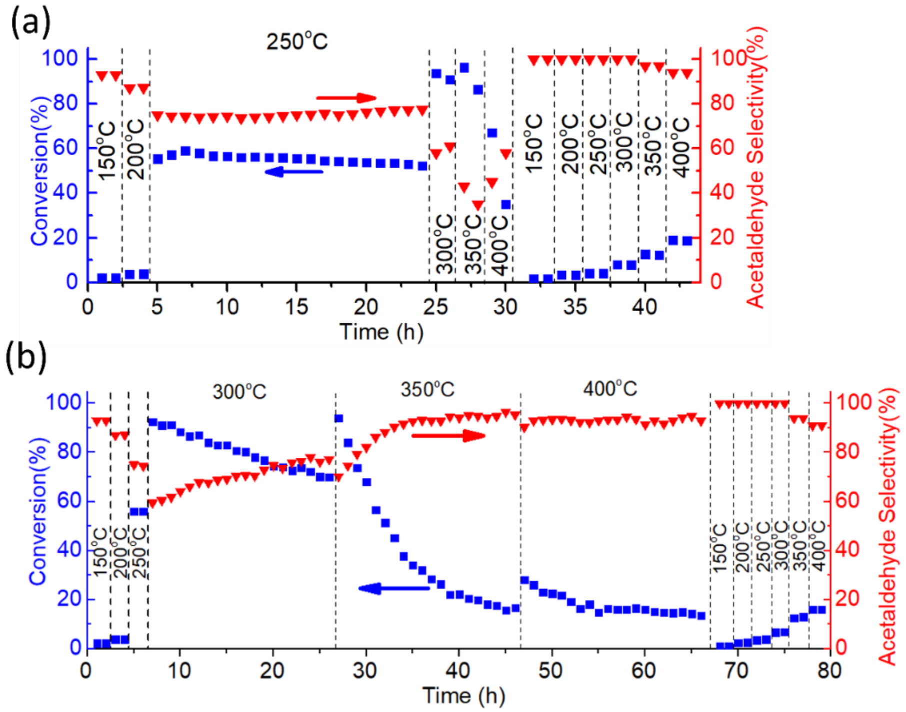

Supplementary Figure 3. (a) Catalytic performance of $Pd_{0.02}Au_{0.98}$ /SiO_2 sample after CO treatment at 30°C for 30 min and 70°C for 30 min. (b) Catalytic performance of a new batch of $Pd_{0.02}Au_{0.98}$ /SiO_2 sample after CO treatment at 30°C for 30 min and 70°C for 30 min. Reaction conditions: 300 mg catalyst, 2 wt.% ethanol in He, total flow rate of 12 mL/min, GHSV= 2400 mL/(h·g_{cat}). Every point is the average conversion/selectivity for 1 h. Each temperature is held for 2 h or 20 h.

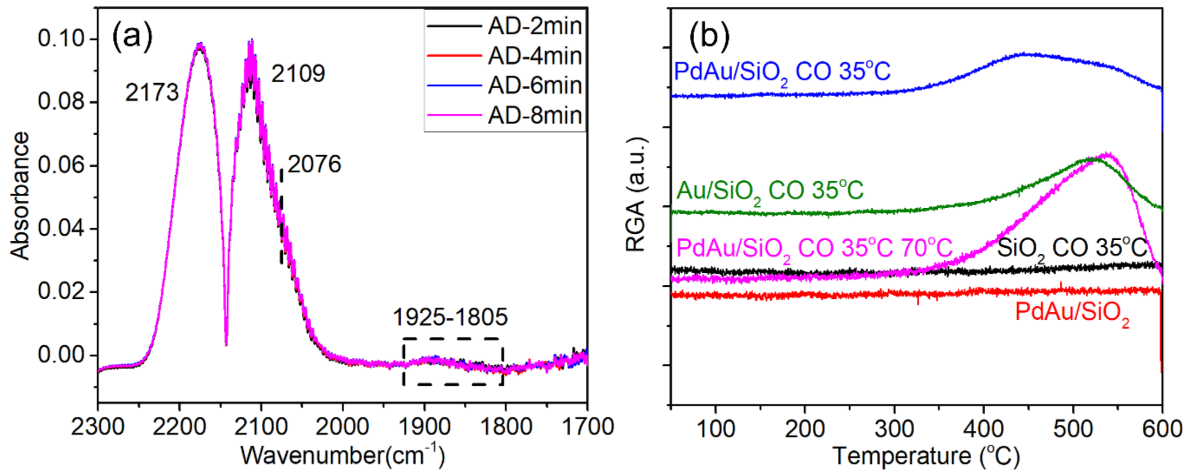

Supplementary Figure 4. (a) CO-DRIFTS results from the $Pd_{0.02}Au_{0.98}$ /SiO_2 sample at 160°C after CO treatment at 30°C for 30 min, 70°C for 30 min, and 30°C for 1h, (b) TPO results of CO_2 signal from samples after various treatments as indicated in the plot. No H_2O was detected.

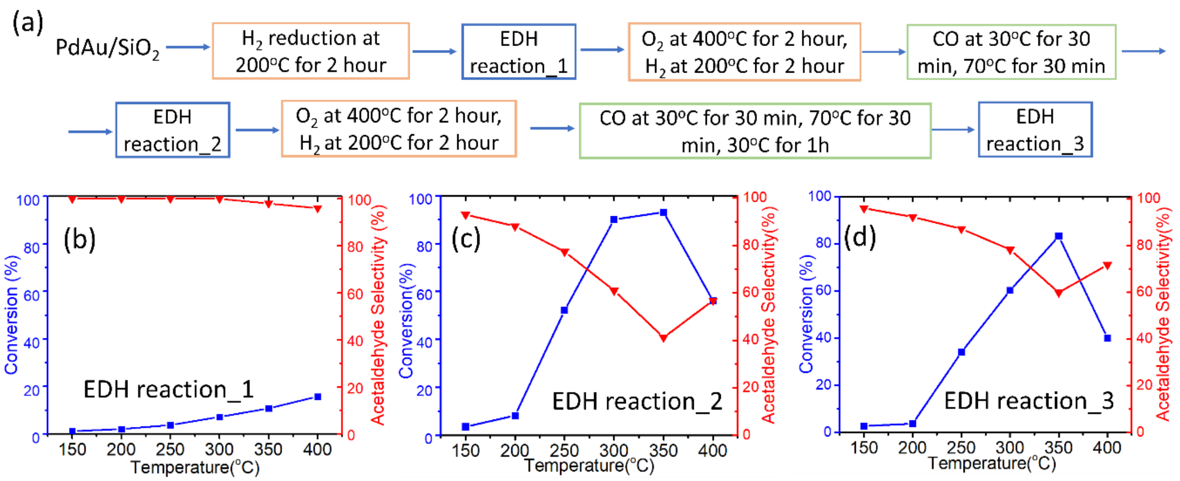

Supplementary Figure 5. The catalytic performance of $Pd_{0.02}Au_{0.98}/SiO_2$ for EDH (b) (c) and (d) after various treatments shown in the reaction pathway as a function of CO pre-treatment in the panel a schematic. Reaction conditions: 300 mg catalyst, 2% ethanol in helium, total flow rate is 12 mL/min, GHSV= 2400 mL/(h·g_{cat}). Each temperature was held for 2 h.

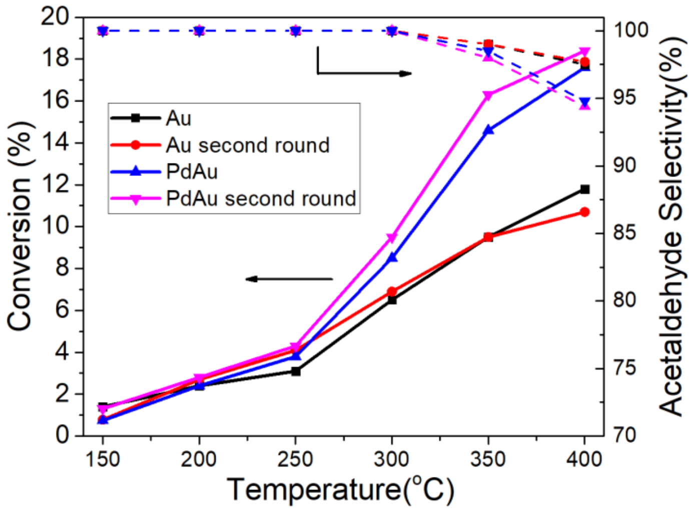

Supplementary Figure 6 Comparison of catalytic performance of Au/SiO_2 and $Pd_{0.02}Au_{0.98}/SiO_2$ after H_2 reduction. Reaction conditions: 300 mg catalyst, 2% ethanol in helium, total flow rate 12 mL/min, GHSV= 2400 mL/(h·g_{cat}). Each temperature was held for 2 h.

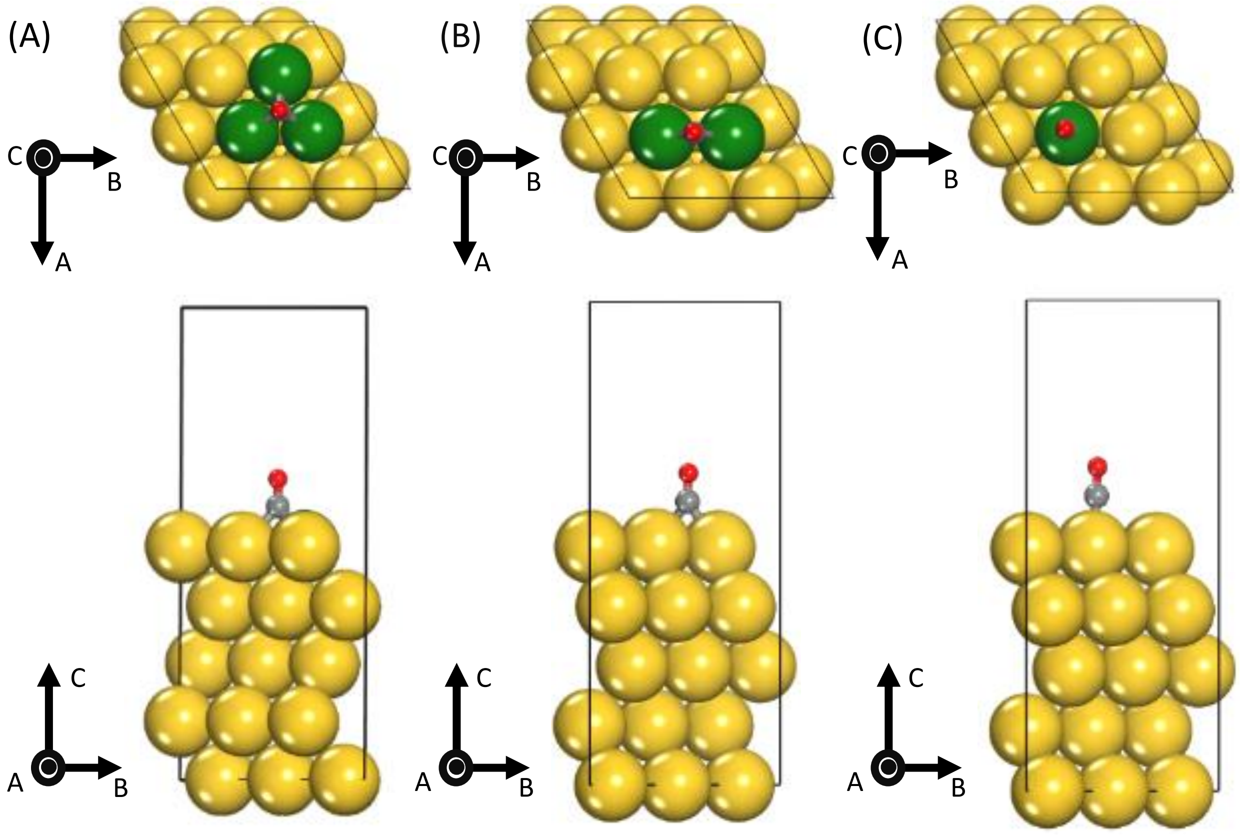

Supplementary Figure 7. Top and side views of the PdAu slab employed for the DFT calculations for (A) a Pd trimer; (B) a Pd-Pd dimer; and (C) a Pd monomer. In all panels the CO adsorption structure on the most stable adsorption site is shown. Au, Pd, C and O atoms are shown in yellow, green, grey and red, respectively. In the schematics of the axes the symbol $\odot$ denotes an arrow pointing from the page towards the reader.

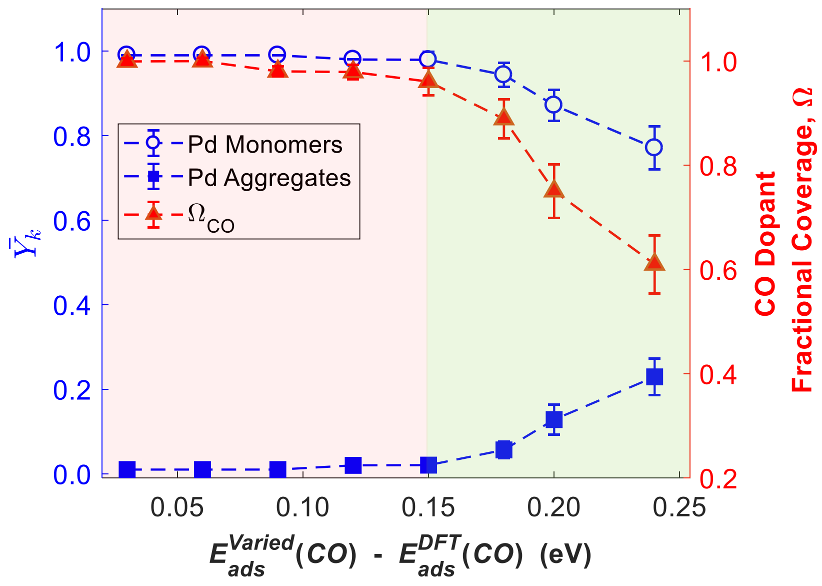

Supplementary Figure 8. Fractions of surface Pd species ($\overline{Y}_k$) and CO dopant fractional coverage (Ω_{CO}) at various smaller binding energies than the DFT-computed value and at 70°C and P_{CO} = 0.1 bar. Almost no Pd aggregates are observed in the red-shaded region, whereas such aggregates are present in the green-shaded. The error bars are ±1 standard deviation.

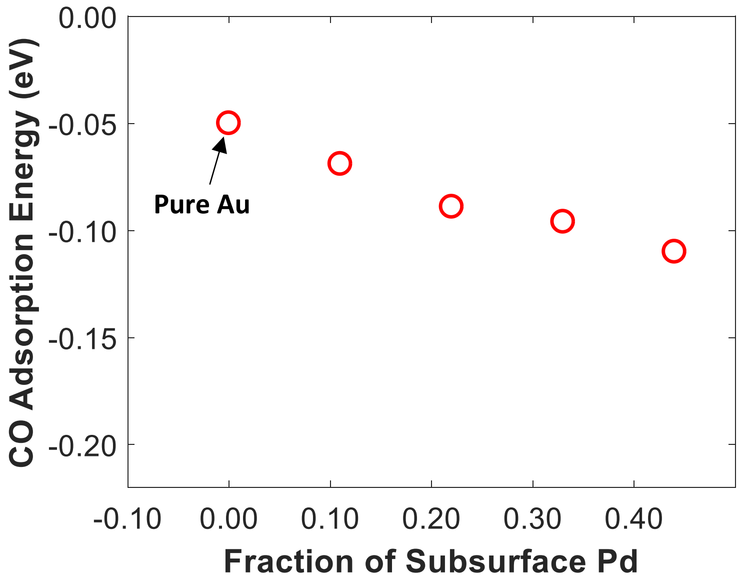

Supplementary Figure 9. Effect of subsurface Pd on the binding strength of CO on Au(111) surfaces.

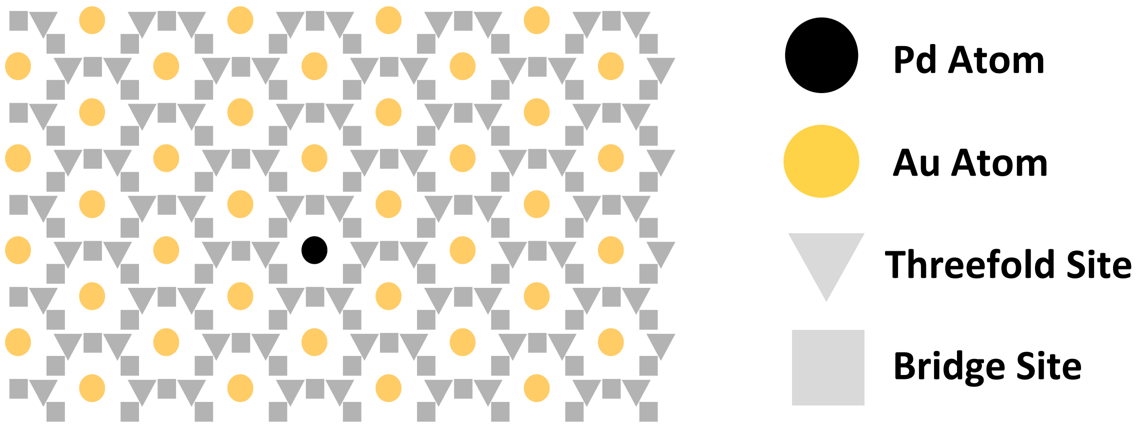

Supplementary Figure 10. A representation of the MC lattice.

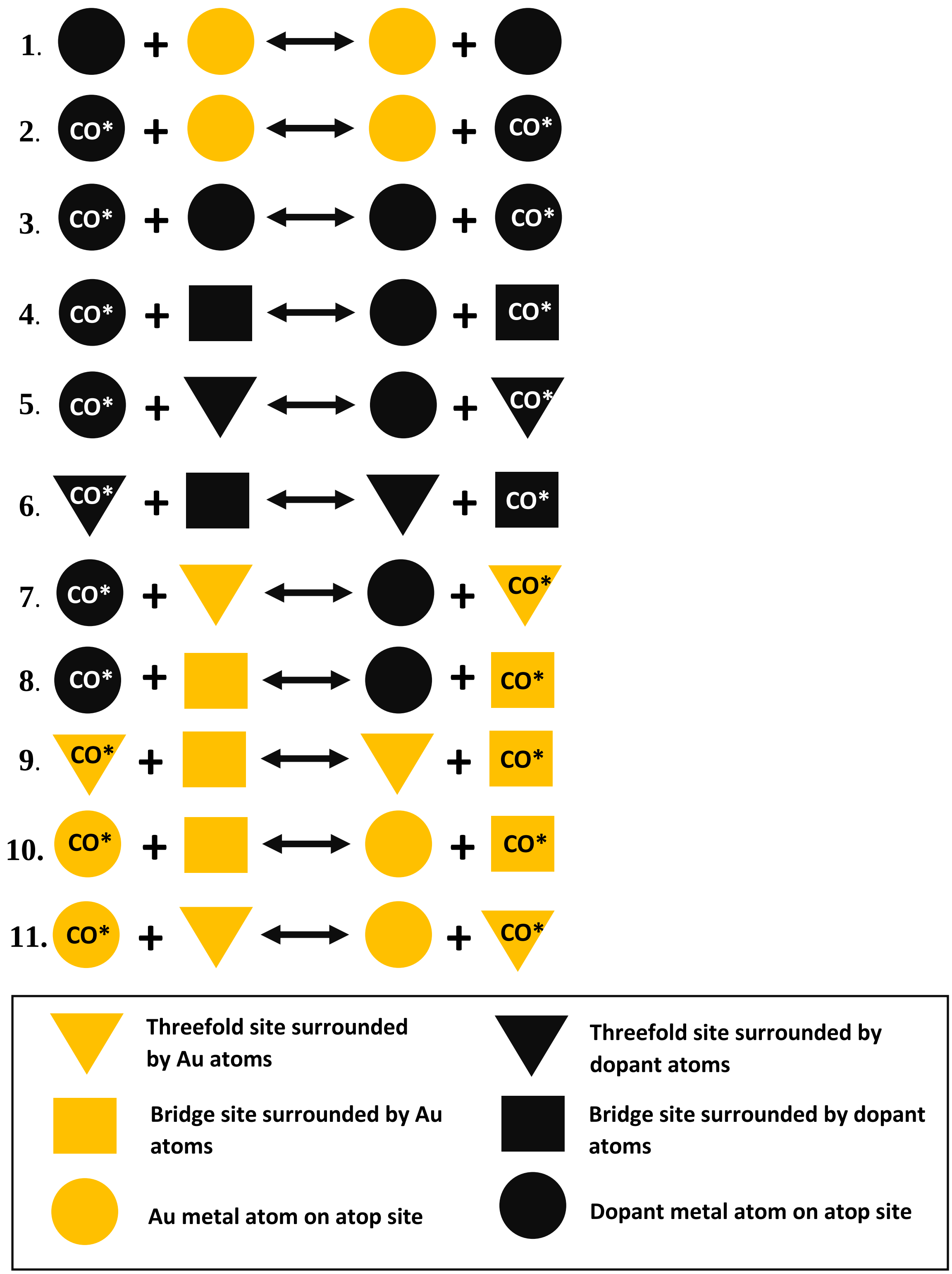

Supplementary Figure 11. Processes 1-3 are atomic swaps between Au and Pd atoms; processes 4-11 are diffusions of CO. The site types that are shown in the reactions are explained in a legend at the bottom of the figure.

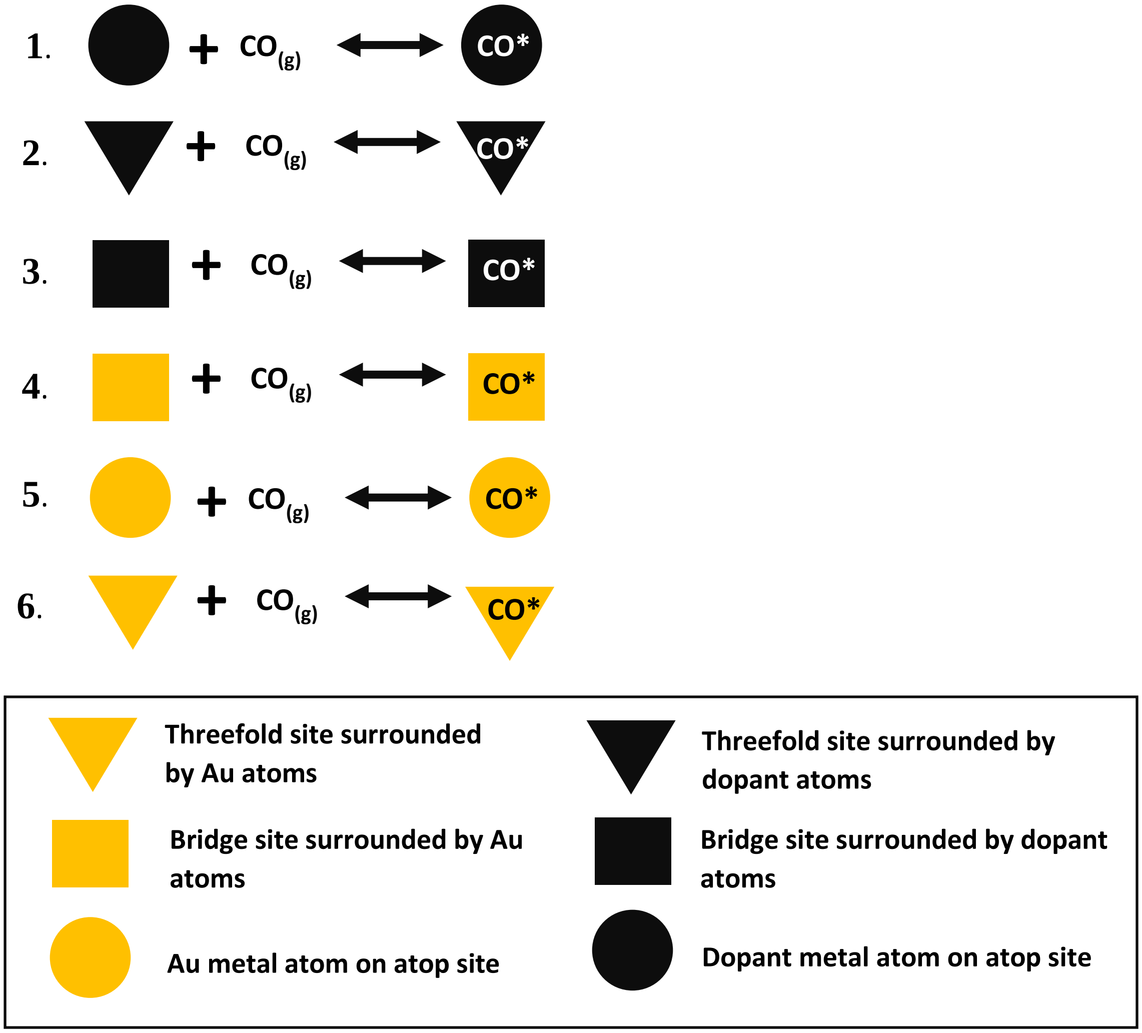

Supplementary Figure 12. CO adsorption/desorption processes that can happen in the Monte Carlo simulation. The site types that are shown in the reactions are explained in a legend at the bottom of the figure.

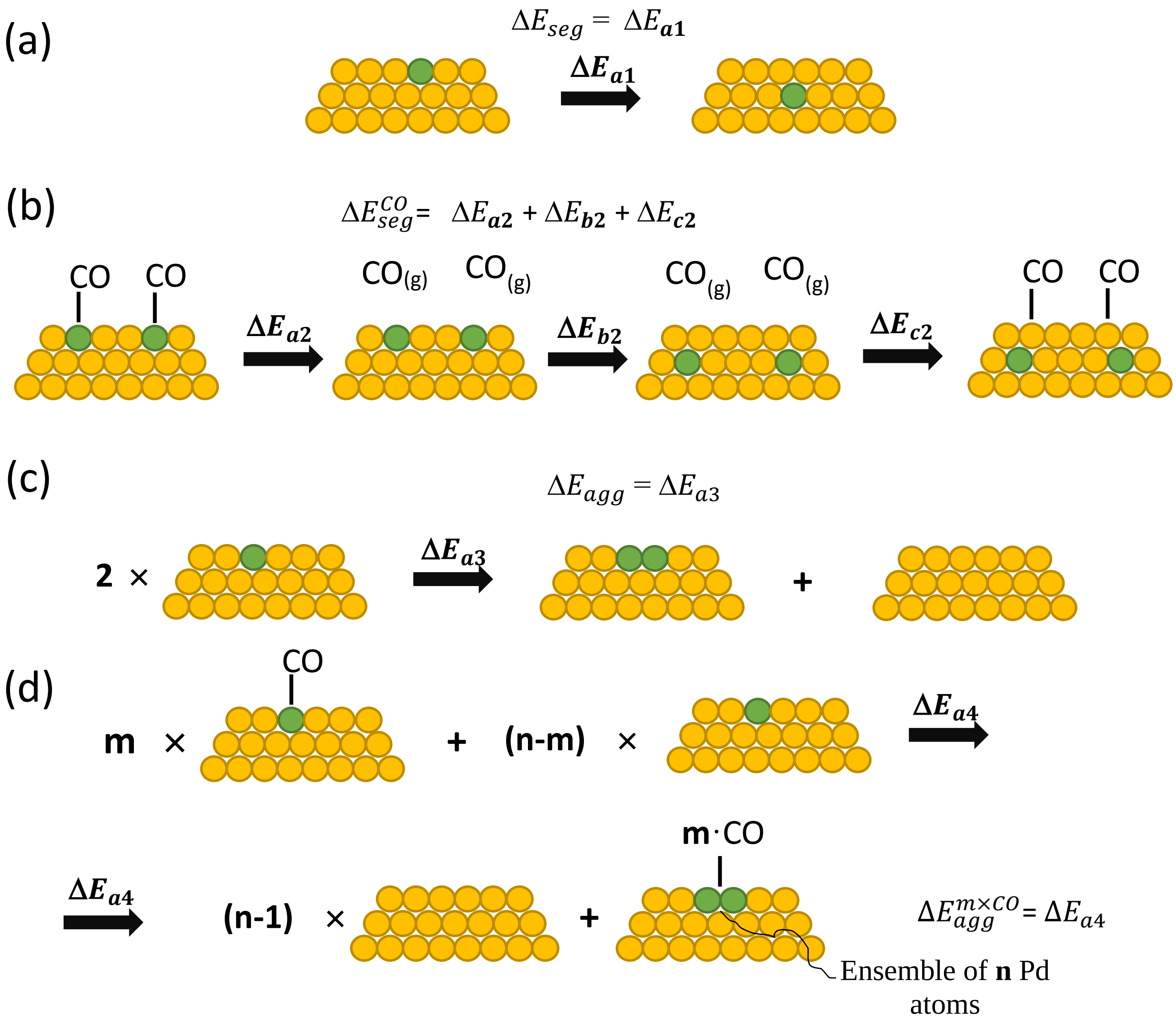

Supplementary Figure 13. Calculation of (a) segregation energy under vacuum; (b) segregation energy under a CO atmosphere; (c) aggregation energy under vacuum; (d) aggregation energy in the presence of CO. Au and Pd atoms are shown in yellow and green, respectively. Note that the number of atoms of each species is always balanced between the final and initial states.

Supplementary Table 1. Metal loading of the samples

Sample	Pd loading (wt.%)	Au loading (wt.%)	Pd/Au atomic ratio
$Pd_{0.02}Au_{0.98}/SiO_2$	0.04	3.5	1/49
Au/SiO_2	-	3.6	-

Supplementary Table 2. Summary of Pd surface species at various simulation temperatures.

$T_{simulation}$ (ºC)	$\overline{Y_{Monomers}}$	$\overline{Y_{Dimers}}$	$\overline{Y_{Trimers}}$	$\overline{Y_{multimers}}$	$\overline{Y_{clusters,total}}$
**30**	0.99	0.01	0.00	0.00	0.01
**34**	0.98	0.02	0.00	0.00	0.02
**38**	0.96	0.03	0.01	0.00	0.04
**42**	0.95	0.04	0.01	0.00	0.05
**46**	0.94	0.05	0.01	0.00	0.06
**50**	0.93	0.05	0.02	0.00	0.07
**54**	0.93	0.06	0.01	0.00	0.07
**58**	0.90	0.07	0.03	0.00	0.10
**62**	0.90	0.08	0.02	0.00	0.10
**66**	0.89	0.09	0.02	0.00	0.11
**70**	0.87	0.10	0.02	0.01	0.13

References

1. Nolin, B. & Jones, R. N. The Infrared Absorption Spectra of Deuterated Esters: I. Methyl Acetate. *Can. J. Chem.* **34**, 1382-1391 (1956).
2. Kumar, A. *et al.* In situ DRIFTS Studies on Cu, Ni and CuNi catalysts for Ethanol Decomposition Reaction. *Catal. Lett.* **146**, 778-787 (2016).
3. Sushkevich, V. L., Ivanova, I. I. & Taarning, E. Mechanistic Study of Ethanol Dehydrogenation over Silica-Supported Silver. *ChemCatChem* **5**, 2367-2373 (2013).
4. Ward, T. *et al.* Effects of Pd on Catalysis by Au: CO Adsorption, CO Oxidation, and Cyclohexene Hydrogenation by Supported Au and Pd–Au Catalysts. *ACS Catal.* **3**, 2644-2653 (2013).
5. Zhu, B. *et al.* Evidence of Pd segregation and stabilization at edges of AuPd nano-clusters in the presence of CO: A combined DFT and DRIFTS study. *J. Catal.* **308**, 272-281 (2013).
6. Luneau, M. *et al.* Enhancing catalytic performance of dilute metal alloy nanomaterials. *Commun. Chem.* **3**, 46 (2020).
7. Xu, J. *et al.* Biphasic Pd−Au Alloy Catalyst for Low-Temperature CO Oxidation. *J. Am. Chem. Soc.* **132**, 10398-10406 (2010).
8. Velasco-Velez, J. J. *et al.* The Role of Adsorbed and Subsurface Carbon Species for the Selective Alkyne Hydrogenation Over a Pd-Black Catalyst: An Operando Study of Bulk and Surface. *Top. Catal.* **61**, 2052-2061 (2018).
9. van Spronsen, M. A. *et al.* The Dynamics of Surface Alloys: Rearrangement of Pd/Ag(111) Induced by CO and O_2. *J. Phys. Chem. C* **123**, 8312-8323 (2019).
10. Enache, D. I. *et al.* Solvent-Free Oxidation of Primary Alcohols to Aldehydes Using Au-Pd/TiO_2 Catalysts. *Science* **311**, 362 (2006).
11. Wrasman, C. J. *et al.* Synthesis of Colloidal Pd/Au Dilute Alloy Nanocrystals and Their Potential for Selective Catalytic Oxidations. *J. Am. Chem. Soc.* **40**, 12930-12939 (2018).
12. Garcia, C. *et al.* Dynamics of Pd Dopant Atoms inside Au Nanoclusters during Catalytic CO Oxidation. *J. Phys. Chem. C* **124**, 23626-23636 (2020).
13. Gautier, S., Steinmann, S. N., Michel, C., Fleurat-Lessard, P. & Sautet, P. Molecular adsorption at Pt(111). How accurate are DFT functionals? *Phys. Chem. Chem. Phys.* **17**, 28921-28930 (2015).
14. Zhu, B., Meng, J. & Gao, Y. Equilibrium Shape of Metal Nanoparticles under Reactive Gas Conditions. *J. Phys. Chem. C* **121**, 5629-5634 (2017).
15. Papanikolaou, K. G., Darby, M. T. & Stamatakis, M. CO-Induced Aggregation and Segregation of Highly Dilute Alloys: A Density Functional Theory Study. *J. Phys. Chem. C* **123**, 9128-9138 (2019).
